# World Hypertension League perspective on public health initiatives to reduce global sodium consumption: are we not seeing the elephant in the room?

**DOI:** 10.1038/s41371-025-01075-9

**Published:** 2025-10-14

**Authors:** Francesco P. Cappuccio

**Affiliations:** 1 University of Warwick, Warwick Medical School, Warwick Applied Health, Coventry, UK; 2 University Hospitals Coventry & Warwickshire NHS Trust, Coventry, UK

**Keywords:** Cardiovascular diseases, Risk factors

## Abstract

Sodium reduction is a well-established strategy for the prevention of cardiovascular disease (CVD), yet effective implementation requires context-specific approaches. Low-sodium salt substitutes (LSSS), in which sodium chloride is partially replaced with potassium chloride, have been investigated as an adjunct intervention. Evidence from randomized controlled trials in Peru and China demonstrates substantial increases in potassium intake and corresponding improvements in blood pressure and CVD outcomes. However, reductions in sodium intake were modest or absent, suggesting that the observed benefits are mediated primarily by potassium rather than sodium reduction. Additional concerns include compensatory sodium consumption from alternative sources, limited evaluation beyond high-risk cohorts, and uncertain safety in populations excluded from trials, such as individuals with kidney disease, children, and pregnant women. Current World Health Organization guidance remains ‘conditional’, reflecting these uncertainties. While LSSS may contribute to CVD prevention strategies, the central objective of global policy continues to be the sustained reduction of population sodium intake.

The article by Egan BM et al. [1] is a timely and valuable reminder of the overwhelming evidence supporting reduction of sodium consumption to prevent cardiovascular disease (CVD) and of the multifaceted initiatives needed for effective public health policies. The authors rightly highlight both the health and economic benefits of reducing sodium consumption, as well as the opportunity costs of failing to act or implementing ineffective policies.

A single, universal policy will not suffice. High Income Countries (HICs) and Middle-Income Countries (MICs) undergoing rapid economic transition and global marketization of food must focus on reducing sodium intake from processed foods and meals eaten outside the home. In contrast, in many Low-and-Middle Income Countries (LMICs) most sodium comes from discretionary use, making these populations more suitable for behavioural health promotion.

Recently, the use of low-sodium salt substitutes (LSSS), which replace sodium chloride, NaCl, with potassium chloride, KCl, has been promoted as an additional tool to help reduce sodium intake. These substitutes have the added benefit of increasing potassium intake, which is known to lower blood pressure and improve CVD outcomes [2], especially in areas where potassium intake is typically low. Recent randomized clinical trials in Peru [3] and China [4, 5] have shown that LSSS can significantly reduce blood pressure [3] and lower rates of fatal and non-fatal CVD events [4, 5].

Egan et al. [1] suggest that replacing 20% of regular salt with a LSSS (75% NaCl, 25% KCl) could reduce sodium intake by 5% per year. Is this assumption supported by evidence? Would the benefits seen in clinical trials translate into real-world effectiveness?

In the Peruvian study [3], the intervention group saw a 32% increase in potassium intake (630 mg/day, 95% CI: 470 to 780), but sodium intake remained virtually unchanged (100 mg/day, 95% CI -230 to 250), representing only a 0.83% reduction per year in a population consuming on average 3,950 mg/day. Similarly, in the Chinese trial [4], potassium intake increased by 57% (800 mg/day, 95% CI 710 to 900), but sodium intake decreased by just 1.7% per year (350 mg/day, 95% CI 150 to 540), a reduction from 4,300 to 3,950 mg/day. Similar results are reported in the sub-study on recurrent stroke [5]. These results suggest that the observed health benefits are likely due to increased potassium intake rather than a significant reduction in sodium. Evidence also suggests a possible beneficial direct vascular effects (independent of blood pressure) [6] that can explain the prevention of stroke recurrence, as seen in the latest Chinese sub-study, in the absence of BP effects [5].

Moreover, since LSSS would theoretically reduce sodium intake by at least a third, the modest reductions observed in trials imply that participants may have compensated by consuming more sodium from other sources. As the use of mono-sodium glutamate (MSG) is predominant in Chinese cooking, it is possible that LSSS might have had a lesser impact on sodium intake not deriving from sodium chloride.

This is the “elephant in the room”: there is currently no convincing evidence that LSSS meaningfully reduces overall sodium consumption. Additionally, the available evidence is limited to high-risk groups (such as hypertensive individuals [3] and older adults with pre-existing CVD [4, 5]) in only two countries. This raises important questions about the safety and generalizability of LSSS for the wider population [7], especially since clinical trials excluded people with kidney impairments, children, and pregnant women [8]. As a result, the World Health Organization currently offers only cautious, conditional recommendations for the use of LSSS and does not directly endorse them for food reformulation [9].

The core principle of global policy remains the reduction of sodium intake [10]. While LSSS may offer some benefits, their role should be considered carefully, with attention to safety, feasibility, and the specific needs of different populations.
